# Enhanced Surface Properties of the Al_0.65_CoCrFeNi High-Entropy Alloy via Laser Remelting

**DOI:** 10.3390/ma16031085

**Published:** 2023-01-26

**Authors:** Junwei Miao, Tianxin Li, Qiang Li, Xiaohu Chen, Zheng Ren, Yiping Lu

**Affiliations:** 1 Key Laboratory of Solidification Control and Digital Preparation Technology (Liaoning Province), School of Materials Science and Engineering, Dalian University of Technology, Dalian 116024, China; 2 Jiangsu XCMG Construction Machinery Research Institute Co., Ltd., Xuzhou 221004, China; 3 Ningbo Branch of China Ordnance Academy, Ningbo 315103, China

**Keywords:** high-entropy alloy, laser remelting, nanoindentation, surface modification, tribological properties

## Abstract

The laser remelting technique was applied to the surface modification of the Al_0.65_CoCrFeNi high-entropy alloy (HEA) to further advance its mechanical potential. The microstructure of the remelted layer was refined from coarse dendritic to submicron-scale basket weave compared with the as-cast substrate, resulting in a 1.8-time increase in Vickers microhardness. The nanoindentation tests indicated that the nanohardness of the remelted layer was higher than that of each phase in the substrate. Meanwhile, the remelted layer retained considerable plasticity, as evidenced by its high *W*_p_/*W*_t_ ratio (0.763) and strain hardening exponent (0.302). Additionally, adhesive wear prevailed on the substrate, while only abrasive wear features were observed on the remelted layer. Accordingly, the average friction coefficient and the wear rate of the remelted layer were minimized by 23% and 80%, respectively, compared with the substrate. Our findings explored an industrialized method to enhance the surface properties of the Al_0.65_CoCrFeNi HEA and also provided some helpful references for its laser additive manufacturing.

## 1. Introduction

High-entropy alloys (HEAs) belong to a newly developed class of alloy materials and contain at least five elements with equal or nearly equal concentrations [1,2,3]. Owing to the unique high-entropy effect and significant multi-principal element effects (i.e., cocktail, lattice distortion, and delayed diffusion), HEAs show many special physical and chemical properties and provide a wealth of new options to meet diverse application scenarios [2,3,4,5]. Nevertheless, the poor wear resistance of some HEAs lowers their competitiveness as advanced structural materials [6,7]. Heat treatment and surface modification are the most common strategies to improve the tribological properties of HEAs with a given composition. For example, Kong et al. [8] found that the AlCoCrFeNiTi_0.5_ HEA annealed at 800 °C for 5 h had a lower friction coefficient and better wear resistance than the as-cast state. Hou et al. [9] applied the plasma nitriding process to modify the surface of Al*_x_*CoCrFeNi HEAs, resulting in a 4- to 18-fold increase in wear resistance. Chen et al. [10] have employed the powder-packed boronizing method for surface strengthening of CoCrNi-based HEA. The results showed that the surface hardness of the boronized specimen was up to 1056 HV, which was three-fold of the untreated specimen. Additionally, a reduction of 63% in wear rate was achieved for the boronized HEA. However, the above methods are often time-consuming, environmentally unfriendly, and equipment dependent, and thus are limited for the widespread application [8,9,10]. 

Laser remelting (LR) is a new surface modification technique that emerged in recent decades with the development of high-power lasers. This technique has some inherent advantages, such as simple process, flexible operation, and economic efficiency, and is already used in conventional alloys, such as Fe [11], Al [12], Mg [13], and Ti [14]. Erdogan et al. [15] studied the effect of LR on the surface structure and properties of electric current assistive sintered CoCrFeNiAl_x_Ti_y_ HEAs. They found that LR refined the alloy grain, eliminated oxide impurity phase and promoted hard BCC phase, which ultimately enhanced hardness and wear resistance of the HEAs. Han et al. [16] performed LR treatment on an as-cast AlCrFe_2_Ni_2_ HEA, and found that both the hardness and yield strength of the remelted alloy were improved remarkably without embrittlement. Cai et al. [17] developed a NiCrCoTiV HEA coating by combining laser cladding and LR techniques. The hardness of the as-remelted coating was enhanced by 200 HV compared to the as-cladded coating. Additionally, the wear volume of the former was less than half of the latter. Recently, Li et al. [18] conducted the surface modification of Al_0.5_CoCrFeNiSi_0.25_ HEA using LR process with different scanning rates. The results showed that both the hardness and wear resistance of HEA enhanced with increasing the laser scanning rate (i.e., solidification rate). To sum up, LR treatment is a promising solution for improving the surface properties of HEAs [16,17,18,19]. 

Al*_x_*CoCrFeNi (*x* is the atomic ratio) is one of the most widely studied HEA systems. The microstructure of the Al*_x_*CoCrFeNi HEAs tends to evolve from a single FCC phase to a mixture of FCC + BCC phases, and further to the BCC/B2 phase, with the increase in the Al content [20,21,22]. A good strength–plasticity combination can be obtained when a soft FCC phase and a hard BCC/B2 phase coexist (0.4 ˂ *x* ˂ 0.9) [20,21,23,24]. However, previous studies found that the wear resistance of as-cast Al*_x_*CoCrFeNi HEAs was poor [25,26,27]. To optimize the tribological properties of the HEAs for their practical application, LR technique was applied for the first time to the surface modification of an as-cast Al_0.65_CoCrFeNi HEA in this study. The microstructure evolution and mechanical and tribological behaviors of the HEAs were investigated systematically. It is envisioned that this study explores an effective approach to enhance the surface properties of the Al*_x_*CoCrFeNi HEA. In addition, our findings might provide the theoretical and technical basis for the laser additive manufacturing of the Al*_x_*CoCrFeNi HEA due to similar working principles.

## 2. Experimental

### 2.1. HEA Preparation and LR Process

The master ingot with a nominal composition of Al_0.65_CoCrFeNi was fabricated from commercially pure (>99.5 wt.%) elements within a protective argon atmosphere. The raw materials with a total weight of 2.3 kg were melted using a medium-frequency induction furnace, then solidified in a high-purity graphite crucible. The obtained cast ingot was first processed into sheets measuring 110 mm × 35 mm × 10 mm, following which the surfaces were ground to 800-grit SiC abrasive paper. A TruDisk 4002 disk laser was used for the LR experiment. Additionally, the laser power, laser spot size, and scanning rates were 4 kW, 30 mm × 2 mm, and 5 mm·s^−1^, respectively. 

### 2.2. Microstructure Characterization

The cross-sectional morphologies of the Al_0.65_CoCrFeNi sample after LR were analyzed using a JXA-8530F Plus electron probe microanalysis system (EPMA) equipped with a backscattered electron (BSE) detector. The microstructures were analyzed in detail using a TalosF200X transmission electron microscope (TEM) equipped with an energy-dispersive spectrometer (EDS). A TEM specimen of the as-cast substrate was prepared by ion milling after mechanical grounding down to 50 μm of thickness. The TEM specimen of the remelted layer was prepared using a Helios G4 UK–focused ion beam instrument.

### 2.3. Mechanical Test

**Figure 1:**
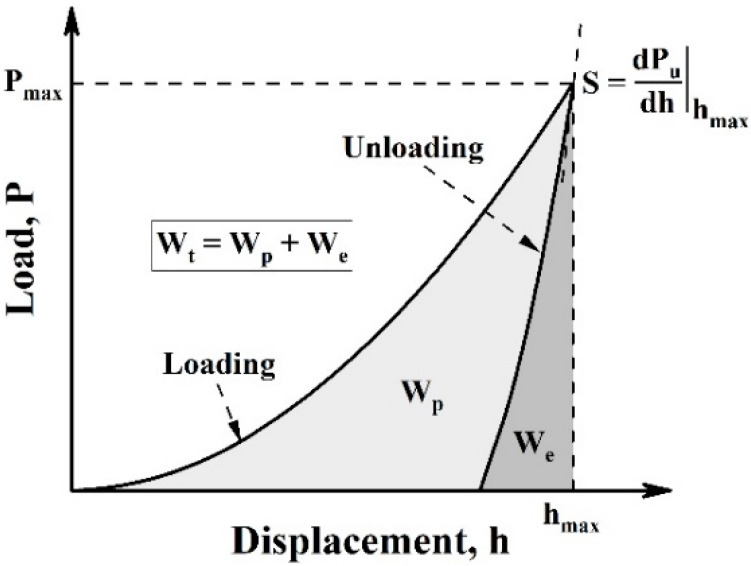
Illustration of a complete load–displacement curve.

The cross-sectional hardness distribution of the LR sample was determined using an MH-50 Vickers microhardness tester. The applied load and duration time were 500 g and 15 s, respectively. Five different indents were measured in each depth region, and the average values were reported. The nanomechanical properties of the LR sample were analyzed using an MTS Nano-Indenter XP system in the following steps: load until the indentation displacement reached 2 μm, hold for 10 s, and unload. Using the Oliver–Pharr method [28], the nanohardness (*H*_n_) was calculated as follows:

(1) $$H_{n}=\frac{P_{\max}}{A_{c}}$$

where *P*_max_ and *A*_c_ are the maximum applied load and the maximum contact projection area, respectively. The elastic modulus (*E*) of the test material was given as:

(2) $$\frac{1}{E_{r}}=\frac{{1\text{−}v}^{2}}{E}+\frac{{1\text{−}v}_{i}^{2}}{E_{i}}$$

where *E*_r_ is the reduced elastic modulus, considering the elastic contributions of the HEAs and indenter. *E*_i_ (1141 GPa) is elastic modulus of the indenter. The *v* (0.25) and *v*_i_ (0.07) are Poisson’s ratio of the HEA and indenter, respectively. The *E*_r_ was calculated as:

(3) $$E_{r}=\frac{\sqrt{\pi }}{2\beta }\frac{S}{\sqrt{A_{c}}}$$

where *S* is the contact stiffness, that is, the slope of the initial part obtained from the unloading curve. *β* is the compensation value of the Berkovich probe (1.034). Figure 1 shows a complete load–displacement (P–h) curve. 

### 2.4. Tribological Test

A HT-1000 ball-on-disk tribometer was employed to evaluate the dry-sliding tribological properties for the as-cast substrate and the laser remelted layer. The Φ 6 mm SiC ball with a hardness of 2800 HV was chosen as the counterpart. The test conditions were as follows: a sliding velocity of 0.28 m·s^−1^, a sliding time of 30 min, and a normal load of 500 g. To ensure the reliability of the data obtained, all tribological tests were repeated at least twice. The friction coefficients were automatically logged by the computer attached to the tribometer. After the tests, the wear tracks of HEAs were examined using MicroXAM-800 three-dimensional (3D) surface profiles. The wear rate was defined as:

(4) $$W=\frac{\Delta V}{S\cdot L}\;$$

where Δ*V* is the wear volume, *S* is the total sliding distance (504 m), and *L* is the normal load (4.9 N). In addition, a Zeiss Supra 55 scanning electron microscope (SEM) equipped with EDS was used to characterize the worn surfaces of HEA disks and SiC balls in order to analyze the wear mechanisms. 

## 3. Results and Discussion

### 3.1. Microstructure Evolution

**Figure 2:**
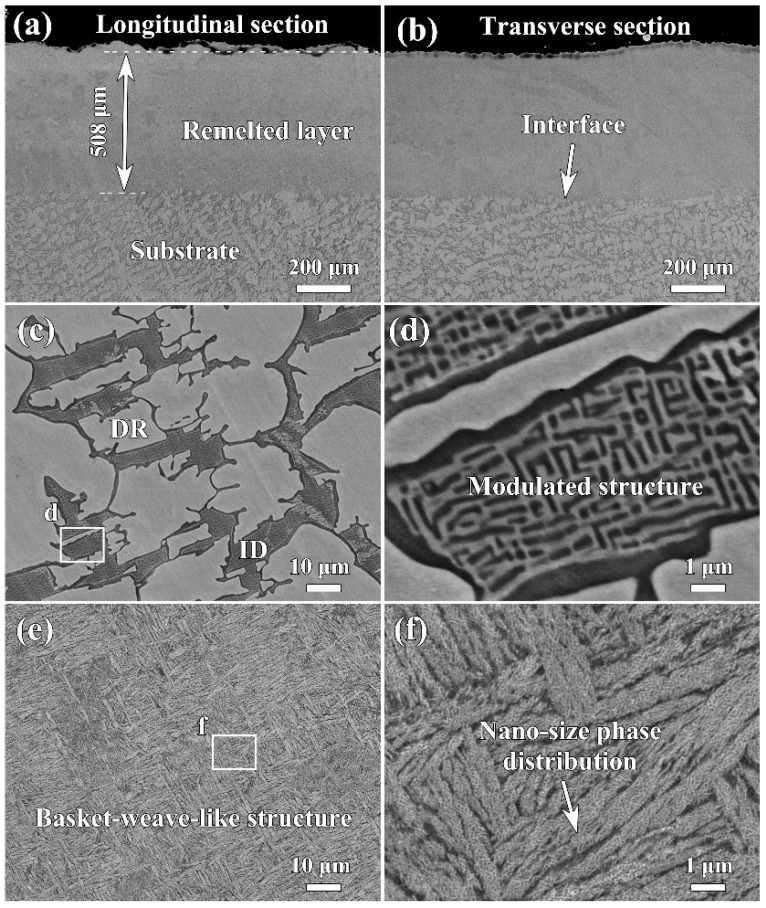
BSE micrographs of the Al_0.65_CoCrFeNi HEA after LR. (**a**) Transverse section; (**b**) longitudinal section; (**c**,**d**) substrate; and (**e**,**f**) remelted layer.

Figure 2 displays the BSE images of the cross-section of the Al_0.65_CoCrFeNi HEA after LR. The low-magnification images showed that the transverse and longitudinal sections had similar morphologies, and both were free of voids and cracks (Figure 2a,b). The laser remelted layer had a thickness of about 508 μm. As shown in Figure 2c, the as-cast substrate exhibited a coarse dendritic structure. It was determined by EPMA point analysis that the chemical compositions of the dendritic (DR) and interdendritic (ID) regions were Al_6.6_Co_21.2_Cr_26.6_Fe_25.1_Ni_20.5_ and Al_16.5_Co_19.4_Cr_18.3_Fe_19.0_Ni_26.8_ (at.%), respectively. The enlarged image in Figure 2c reveals that the DR region was a purely single phase, while the ID region exhibited a maze-like modulation structure consisting of two alternating fine phases (Figure 2d). Similar as-cast morphology has been frequently observed in FCC + BCC (B2 + A2) structured Al*_x_*CoCrFeNi HEAs (0.4 < *x* < 0.9) [20,21,23,24]. During the cooling process, the (Cr, Fe, Co)-rich FCC DR first formed, then the rest elements solidified as a (Al, Ni)-rich BCC phase in the ID region. When the temperature decreased to a critical value, the supersaturated BCC phase further decomposed into two different BCC phases via the spinodal decomposition mechanism [21,24]. Unlike the as-cast substrate, the laser remelted layer exhibited a fine basket weave–like structure, as shown in Figure 2e. Furthermore, the nano-size phase distribution was observed from a high-magnification image (Figure 2f). The EPMA point analysis found that the chemical composition of the remelted layer was extremely close to the nominal composition of the Al_0.65_CoCrFeNi HEA, implying that no significant burnout occurred during the LR process. In other words, the microstructure evolution of the HEA was mainly caused by different solidification conditions.

**Figure 3:**
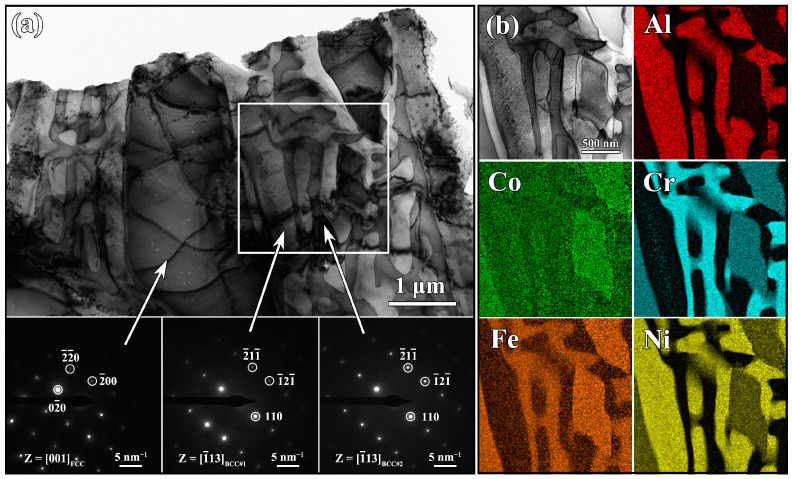
TEM analysis for the substrate. (**a**) BF image and SAED patterns; (**b**) STEM image and EDS elemental maps of the rectangle region marked in (**a**).

The in-depth characterization was conducted using TEM to better reveal the microstructure evolution of the HEA under different solidification conditions. Consistent with the BSE images, the TEM bright-field (BF) images from the as-cast substrate also show a typical dendritic morphology (Figure 3a). By calibrating the SAED patterns, it was confirmed that the DR region was one FCC phase, while the ID region contained two different BCC phases. The lattice constant of three phases was $a_{\mathrm{FCC}\;}=0.3627\text{ nm}$, $a_{\mathrm{BCC}\text{\#}1}=0.2923\text{ nm}$, and $a_{\mathrm{BCC}\text{\#}2}=0.2936\text{ nm}$, respectively. The lattice mismatch of BCC#1 and BCC#2 was only about 0.44%, indicating that the two phases were coherent [29]. Figure 3b shows the scanning TEM (STEM) image of the rectangular region marked in Figure 3a and the corresponding EDS elemental maps. Table 1 lists the chemical composition for the different phases determined by TEM-EDS. The FCC phase was enriched for Cr, Fe, and Co, in good agreement with the EPMA results. The ID region exhibited a distinct compositional separation, where the BCC#1 phase was (Al, Ni)-rich and the BCC#2 phase was (Cr, Fe)-rich. Among the constituent elements of the Al_0.65_CoCrFeNi HEA, the mixing enthalpies between Co, Cr, and Fe were close to zero and their atomic radii were similar; therefore, they tended to form an FCC phase. The Al and Ni elements had a strong combined tendency to form a BCC phase due to their very negative mixing enthalpy (–22 kJ·mol^−1^) [30]. In particular, the BCC phase formed at high temperatures was unstable. When the temperature decreased to a critical value, the initial BCC phase further decomposed into two coherent BCC phases (i.e., BCC#1 and BCC#2) via the spinodal decomposition mechanism [21,24].

**Figure 4:**
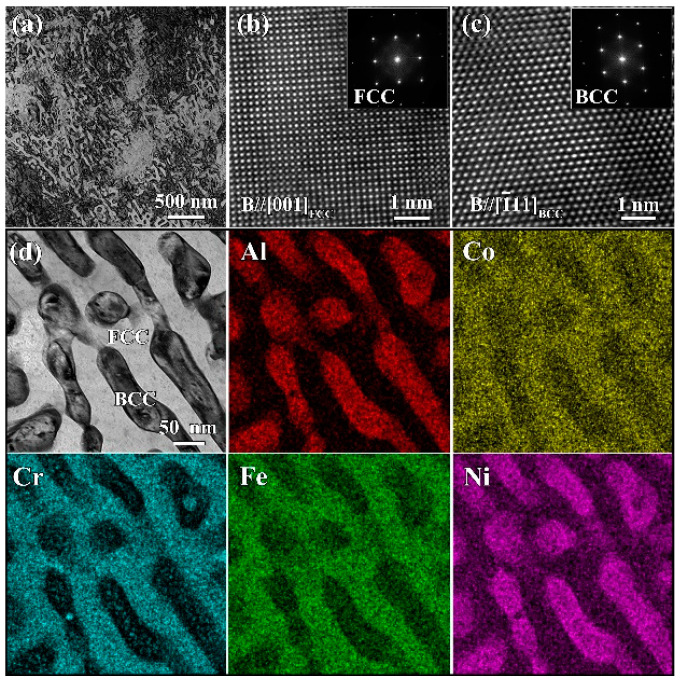
TEM analysis for the remelted layer. (**a**) BF image; (**b**,**c**) HRTEM images and FFT patterns (insets) for the two constituent phases; and (**d**) STEM image and corresponding EDS elemental maps.

Figure 4a shows a TEM-BF image of the laser-remelted layer. Consistent with the BSE images, the microstructure of the remelted layer had a fine weave-like morphology composed of two alternating phases. Figure 4b,c gives the high-resolution TEM (HRTEM) images of the two constituent phases and corresponding fast Fourier transformation (FFT) patterns. It was confirmed that one was the FCC phase, and the other was the BCC phase, and their lattice constants were $a_{\mathrm{FCC}}=0.3590\text{ nm}$ and $a_{\mathrm{BCC}}=0.2870\text{ nm}$, respectively. Figure 4d presents a STEM image and corresponding EDS elemental maps. Table 1 also lists the chemical composition of the two constituent phases. The Al content of the FCC phase in the remelted layer was slightly lower than that of the FCC phase in the substrate, rationalizing the relatively low lattice constant of the former. Only one BCC phase was detected in the remelted layer, and its chemical composition and lattice constant were close to those of the (Al, Ni)-rich BCC#1 phase in the substrate. Compared with the direct casting method, the LR process could provide an ultrafast cooling rate (10^3^ to 10^6^ K·s^−1^) and a large thermal gradient (10^5^ to 10^7^ K·m^−1^), which were conducive to increasing the nucleation rate, suppressing grain growth, eliminating segregation, and forming metastable new phases [16,31]. From the viewpoint of material design, such a fine composite structure consisting of a soft FCC phase and a hard BCC phase is attractive, and the synergistic deformation of the two constituent phases may lead to a good strength–plasticity combination.

### 3.2. Mechanical Properties

**Figure 5:**
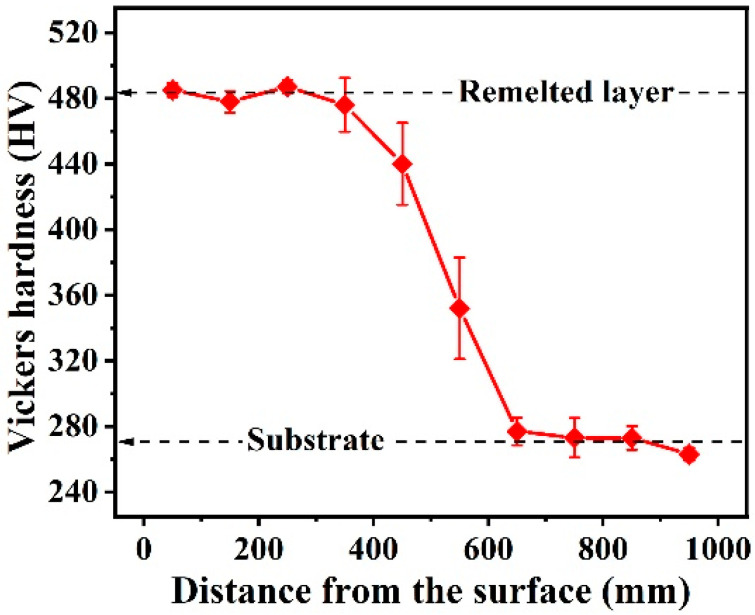
Cross-sectional microhardness distribution of the Al_0.65_CoCrFeNi specimen after LR.

Figure 5 plots the cross-sectional microhardness distribution of the Al_0.65_CoCrFeNi specimen after LR. The average microhardness of the remelted layer was as high as 483 HV, which was enhanced by 78% compared with that of the substrate. The causes for the substantial increase in the microhardness of the remelted layer were threefold: (i) the LR process could provide an ultrafast cooling rate, which increased the nucleation rate and suppressed grain growth, resulting in significant fine-grain strengthening; (ii) the remelted layer had a finely composite structure including FCC and BCC phases, and the abundant phase boundaries impeded the movement of dislocations; and (iii) the volume fraction of the hard BCC phase increased under fast solidification conditions [16,31]. The microstructure of the remelted HEA was so fine that the present study could not accurately measure the volume fraction of the BCC phases.

**Figure 6:**
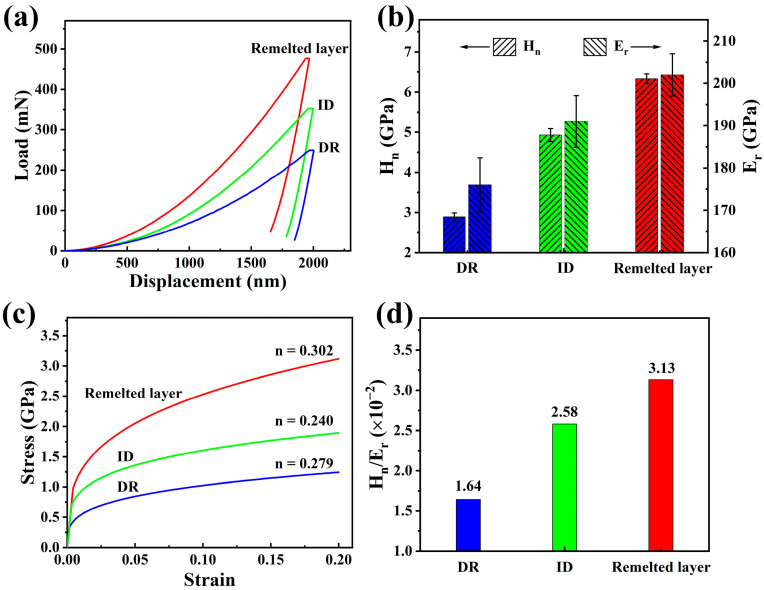
Nanoindentation analysis results. (**a**) Typical P–h curves; (**b**) *H*_n_ and *E*_r_; (**c**) calculated stress–strain curves; and (**d**) *H*_n_/*E*_r_ ratios.

A cross-section specimen was characterized by nanoindentation to fully reveal the effect of LR on the mechanical properties of the Al_0.65_CoCrFeNi HEA. Figure 6a shows the P–h curves of the three feature regions (i.e., DR and ID of the substrate and the remelted layer). The curves were obtained using the depth control mode. According to the Oliver–Pharr method [28], the nanohardness (*H*_n_) and the reduced elastic modulus (*E*_r_) were calculated and summarized in Figure 6b and Table 2. The *H*_n_ of the remelted layer was ~2.2 and ~1.3 times that of the DR region (FCC phase) and ID region (BCC phase), respectively, indicating that the grain/phase boundary strengthening contributed greatly to the high hardness of the remelted layer. The *H*/*E* ratio, associated with the elastic strain to failure, has been shown to be a reliable indicator for predicting the wear resistance of materials. The materials with high H/E ratio often have a good wear resistance. Figure 6d compares the average *H*_n_/*E*_r_ of the three feature regions. The *H*_n_/*E*_r_ ratio of the remelted layer was higher than any region of the as-cast substrate, and, therefore, an enhanced wear resistance of the remelted layer could be expected. As shown in Figure 1, the integral areas of the loading and unloading curves were the total work (*W*_t_) and elastic work (*W*_e_) during indentation process, respectively. The area enclosed by the two curves was the plastic work done in the indentation test, that is, *W*_p_ = *W*_t_ − *W*_e_. The *W*_p_/*W*_t_ ratio can be used to analyze the plasticity of a material qualitatively, and a larger *W*_p/_*W*_t_ ratio indicates that the indented material has better plasticity [16,32]. The *W*_p_/*W*_t_ ratio of the DR and ID of the substrate was calculated as 0.888 ± 0.004 and 0.840 ± 0.003, respectively. The *W*_p_/*W*_t_ ratio of the remelted layer was 0.763 ± 0.003, indicating that the laser remelted HEA maintained considerable plasticity.

Furthermore, the compression stress–strain behaviors of the HEAs can be determined from the P–h curve through a reverse analysis algorithm [32]. The stress–strain relationship of the HEAs was assumed to follow Hooke’s law and power law in the elastic and plastic stages, respectively [33]:

(5) $$\sigma =\{\begin{array}{ll} E\varepsilon & \;\sigma \;\leq {\;\sigma }_{y}\\ \sigma_{y}{\left(1\;+\;\frac{E}{\sigma_{y}}\varepsilon_{p}\right)}^{n} & \;\sigma \;\geq \;\sigma_{y} \end{array}$$

where E is the elastic modulus, ε is the total strain, $\sigma_{y}$ is the initial yield stress at zero offset strain, $\varepsilon_{p}$ is effective strain accumulated beyond yield strain, and *n* is the strain hardening exponent. The detailed calculation steps can be found in references [33,34]. Table 2 summarizes the key parameters obtained by calculation, and Figure 6c represents the corresponding true stress–strain curves. It was seen that the $\sigma_{y}$ of the remelted layer was 0.973 GPa, which was 2.96 and 1.37 times higher than that of the DR and ID regions, respectively. In addition, the *n* value of the remelted layer was larger than those of the DR and ID regions. The HEAs with a large *n* value had a high strain hardening effect, leading to uniform deformation, alleviating stress concentration, and inhibiting cracks. Thus, although the strength and hardness of the Al_0.65_CoCrFeNi HEA were greatly enhanced after LR treatment, a good plasticity was maintained. Owing to the similar working principle, our present study provided new insight for the laser additive manufacturing of the Al_0.65_CoCrFeNi HEA as the raw material.

### 3.3. Tribological Properties

**Figure 7:**
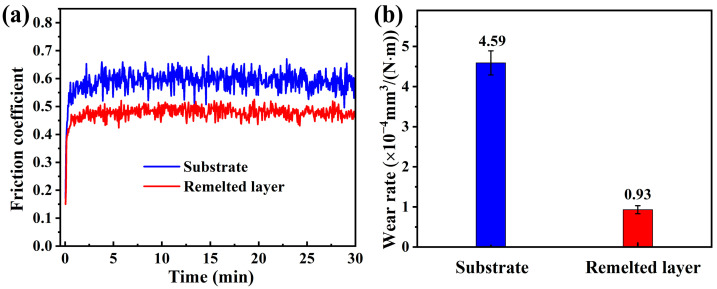
Tribological test results. (**a**) Friction coefficient; and (**b**) wear rates.

Figure 7a displays the typical friction coefficient curves of the substrate and the remelted layer sliding against SiC balls under dry conditions. In both cases, the curves first showed a rapid increase, then stabilized after a running-in period of approximately 60–90 s. However, the average friction coefficient of the substrate was relatively high, reaching 0.59, and the curve fluctuated noticeably. This fluctuation was generally accepted due to the accumulation and removal of wear debris during the test process. The accumulation of wear debris roughened the worn surface, leading to an increase in friction coefficient; when the wear debris were removed, the sliding resistance of the counterpart ball was reduced, and the friction coefficient decreased accordingly. For the remelted layer, the curve was relatively smooth, and the average friction coefficient was only 0.48. In fact, the friction coefficient in dry friction tests can be expressed as [35]:

(6) $$f=\frac{\tau_{b}}{\sigma_{s}}+\frac{2}{\pi }\cot\theta$$

where $\tau_{b}$ and $\sigma_{s}$ are the shear strength and yield strength of the alloy, respectively, and θ is a geometric parameter of the abrasive particles. The first part of Equation (6) is mainly attributed to adhesive wear. After LR, the yield strength of the HEA was obviously enhanced (Figure 6c); therefore, the effect of the adhesive wear was weakened. The second part of Equation (6) is attributed to the contribution of the abrasive wear. The counterpart materials used in this study were the same, implying little difference in the abrasive wear. The sum of the two parts indicated that the remelted layer had a small friction coefficient, which was consistent with the current test results.

Figure 7b compares the wear resistance of the Al_0.65_CoCrFeNi HEA before and after LR. The average wear rate of the substrate and the remelted layer was 4.59 and 0.93 (× 10^−4^ mm^3^/(N·m)), respectively; that is, the latter was only 20% of the former. The wear resistance of materials was linked to their hardness in the classical Archard law [36]. The materials with high hardness had good resistance to plastic deformation, which could weaken the plowing action from the counterface materials, thus reducing the wear rate. As shown in Figure 5, the surface hardness of the HEA after LR increased from the original 271 HV to the final 483 HV. Therefore, it is readily accepted that the remelted HEA has a better wear resistance. Furthermore, Leyland et al. found [37] that the elastic modulus was also critical in determining the wear behavior of materials, and proposed that H/E was a more suitable parameter in predicting wear resistance. As shown in Figure 6d, the remelted layer had a larger *H*_n_/*E*_r_ ratio, further rationalizing its better wear resistance.

**Figure 8:**
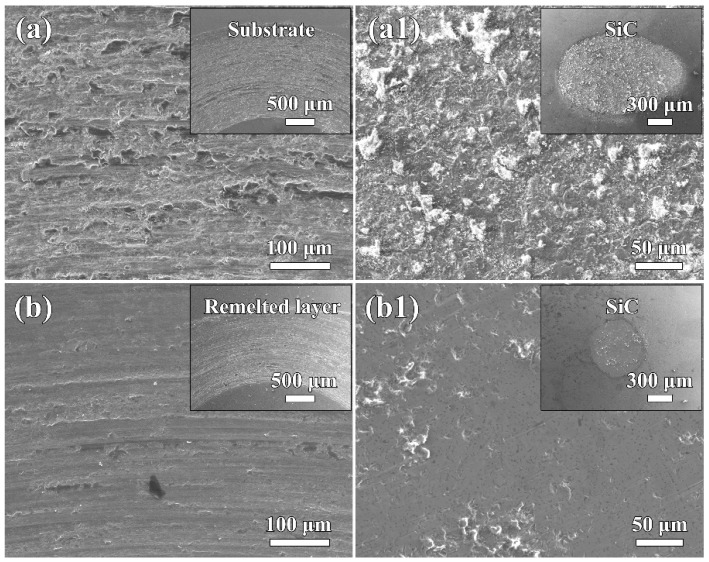
Worn surface morphologies. (**a**)/(**a1**): substrate/SiC; (**b**)/(**b1**): remelted layer/SiC.

Figure 8 presents the SEM secondary electron images of the worn surfaces of the HEAs and their corresponding SiC balls. It can be seen from Figure 8a that the wear track of the as-cast substrate was wide and rough. Owing to the low hardness of the substrate, severe plastic deformation and delamination features occurred on its worn surface. In addition, a substantial number of adhesive patches were found on the worn surface of the SiC ball after sliding against the substrate (Figure 8a1). The average chemical composition of these patches was determined using EDS as Al_4.3_Co_6.9_Cr_6.7_Fe_6.3_Ni_7.8_O_68.0_ (at.%), indicating that the substrate underwent adhesive and oxidative wear. As shown in Figure 8b, the wear track of the remelted layer was significantly narrower. Additionally, only few scattered debris and some parallel grooves were detected on its worn surface. After sliding against the remelted layer, the wear spot size of the SiC ball was small, and its worn surface was smooth and non-adhesive (Figure 8b1). Obviously, the dry wear process for the remelted layer was dominated by the abrasive wear mechanism. The reduction in the adhesive wear allowed the remelted layer to exhibit a lower friction coefficient and wear rate than the substrate (Figure 7).

## 4. Conclusions

In this study, the LR technique has been applied to the surface modification of the Al_0.65_CoCrFeNi HEA for the first time. The microstructure, mechanical and tribological behaviors for the substrate and the remelted layer were investigated comparatively. The main conclusions were as follows:

1. The as-cast Al_0.65_CoCrFeNi HEA exhibited a coarse dendritic structure, in which the dendrite region was identified as the FCC phase, and the interdendritic region was composed of two different BCC phases. Due to the ultra-fast cooling rate of the LR process, the microstructure of the laser remelted layer transformed into a fine basket-weave morphology consisting of the FCC phase and BCC phase.
2. The LR treatment resulted in a significant rise in the surface hardness of the Al_0.65_CoCrFeNi HEA. Specifically, the Vickers microhardness of HEA increased from the original 271 HV to the final 483 HV, with a 78% increment. Our study explores an industrial-ready surface strengthening method for the HEA.
3. The nanoindentation analysis indicated that the laser remelted HEA not only possessed high hardness and strength, but also exhibited a high *W*_p_/*W*_t_ ratio (0.763) and a strain hardening exponent (0.302), implying that it maintained considerable plasticity.
4. The average friction coefficient and the wear rate of the remelted alloy were minimized by 23% and 80%, respectively, compared with that of the as-cast HEA, which was attributed to the reduction in the adhesive wear of the remelted layer.

## Figures and Tables

**Table 1:** TEM-EDS results (at.%) for the different phases in the substrate and remelted layer.

	Al	Co	Cr	Fe	Ni
*Substrate*					
FCC	5.8 ± 0.5	19.6 ± 1.1	26.6 ± 0.3	26.8 ± 0.5	21.2 ± 0.8
BCC#1	24.8 ± 1.5	16.5 ± 0.2	5.4 ± 0.9	13.7 ± 0.7	39.6 ± 0.6
BCC#2	0.7 ± 0.2	15.2 ± 0.6	53.0 ± 0.4	27.1 ± 0.8	4.0 ± 0.9
*Remelted layer*					
FCC	4.0 ± 0.4	21.0 ± 1.5	25.5 ± 0.2	27.6 ± 0.7	21.9 ± 0.9
BCC	20.5 ± 0.9	15.4 ± 0.2	10.8 ± 2.6	16.6 ± 0.9	36.6 ± 2.4

**Table 2:** Nanomechanical properties of the substrate and the remelted layer obtained from the nanoindentation tests.

Regions		*H*_n_ (GPa)	*E*_r_ (GPa)	*E* (GPa)	*σ*_y_ (GPa)	*n*
Substrate	DR	2.89 ± 0.10	176 ± 6	194 ± 3	0.328 ± 0.007	0.279 ± 0.007
	ID	4.93 ± 0.16	191 ± 6	214 ± 4	0.708 ± 0.032	0.240 ± 0.002
Remelted layer		6.33 ± 0.12	202 ± 5	230 ± 7	0.973 ± 0.081	0.302 ± 0.012

## Data Availability

Not applicable.
